# Assembly and Mutagenesis of Human Coronavirus OC43 Genomes in Yeast via Transformation-Associated Recombination

**DOI:** 10.21769/BioProtoc.5422

**Published:** 2025-08-20

**Authors:** Brett A. Duguay, Craig McCormick

**Affiliations:** Department of Microbiology & Immunology, Dalhousie University, Halifax, NS, Canada

**Keywords:** Virus, Coronavirus, Reporter virus, HCoV-OC43, Reverse genetics, Yeast, Transformation-associated recombination, TAR

## Abstract

Human coronavirus OC43 (HCoV-OC43) is an endemic “common cold” coronavirus widely used to study fundamental aspects of coronavirus biology and to test therapeutic interventions. Recently, we used a yeast-based reverse genetics strategy to create recombinant HCoV-OC43 and fluorescent reporter viruses. We assembled a DNA copy of the HCoV-OC43 genome from six linear dsDNA fragments and a linearized yeast centromeric plasmid/bacterial artificial chromosome (YCpBAC) vector in *Saccharomyces cerevisiae* using transformation-associated recombination (TAR). Reporter genes encoding mCardinal fluorescent protein or histone H2B fused to mClover3 (*mClover-H2B*) or mRuby3 (*mRuby-H2B*) were inserted into an intergenic region between the HCoV-OC43 *M* and *N* genes. Assembled full-length HCoV-OC43-encoding plasmids were delivered into permissive mammalian cells to initiate viral gene expression, genome replication, and production of infectious progeny. This technique allows for the precise mutagenesis of any area of the HCoV-OC43 genome using homologous recombination, yielding genetically defined reference plasmids for the future generation of HCoV-OC43 virus stocks.

Key features

• Utilizes the previously developed TAR assembly method [1] to assemble and mutagenize a double-stranded DNA copy of the single-stranded RNA HCoV-OC43 genome [2].

• The availability of multiple sub-genomic and full-length HCoV-OC43-encoding plasmids provides flexibility in how substitution or deletion mutations can be incorporated using PCR or restriction cloning.

• Reporter viruses enable rapid visualization and quantification of infection.

• Generate and isolate a mutagenized HCoV-OC43 plasmid in approximately 14 days, followed by rescue of infectious virus in an additional 12–16 days.

## Graphical overview


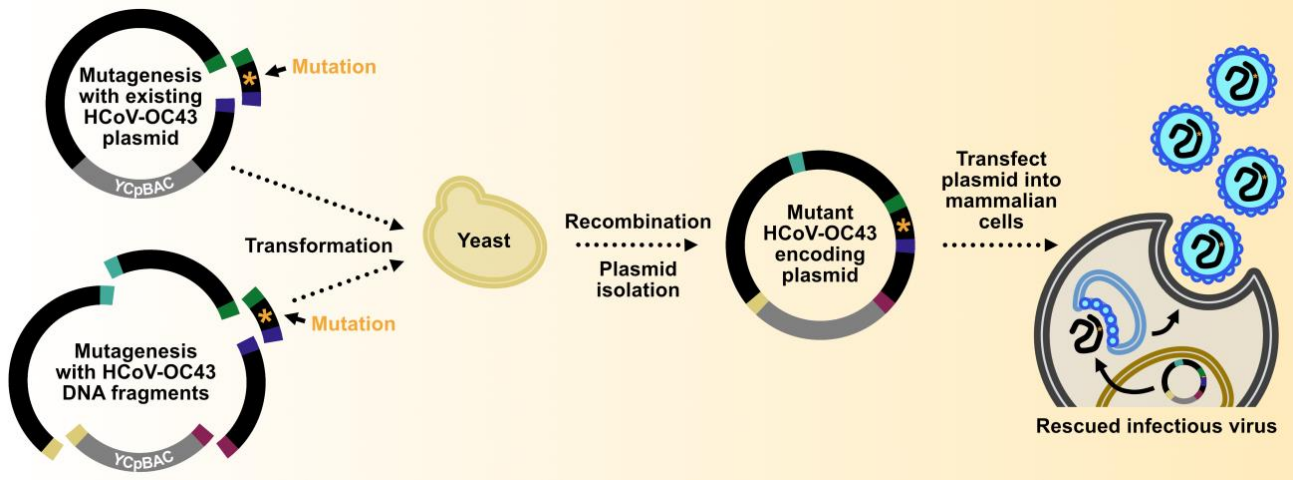


## Background

Viral reverse genetics systems are essential tools for manipulating viral genomes to elucidate fundamental aspects of virus biology, developing reporter viruses to track or measure viral replication, and evaluating antiviral interventions. A common tactic for manipulating large, positive-sense, single-stranded RNA (ssRNA) coronavirus genomes involves encoding genetic information as complementary DNA (cDNA) copies that are more easily manipulated and propagated in vitro. Multiple techniques have been used to generate intact, full-length DNA copies of coronavirus genomes, including Vaccinia virus vectors [3–6], bacterial artificial chromosomes (BACs) [7–15], and, most recently, yeast episomal or centromeric plasmids [2,16–21].

Using *Saccharomyces cerevisiae*, large DNA plasmids encoding entire viral genomes are assembled from smaller double-stranded (dsDNA) fragments via homologous recombination using a technique known as transformation-associated recombination (TAR). This allows for larger viral genomes to be broken down into more manageable fragments amenable to DNA synthesis or standard mutagenesis techniques. Provided that adjacent dsDNA fragments share ~50 bp of homologous sequence, fragments will be stitched together using the efficient DNA recombination machinery in *S. cerevisiae*. Yeast assembly of viral genomes began with the cloning of the 5.4 kb bacteriophage øX174 genome [22]. Since then, this technique has been successfully applied to multiple RNA and DNA viruses, with the largest viral assembly (235 kbp) completed for the human cytomegalovirus genome [23].

**Figure 1.**
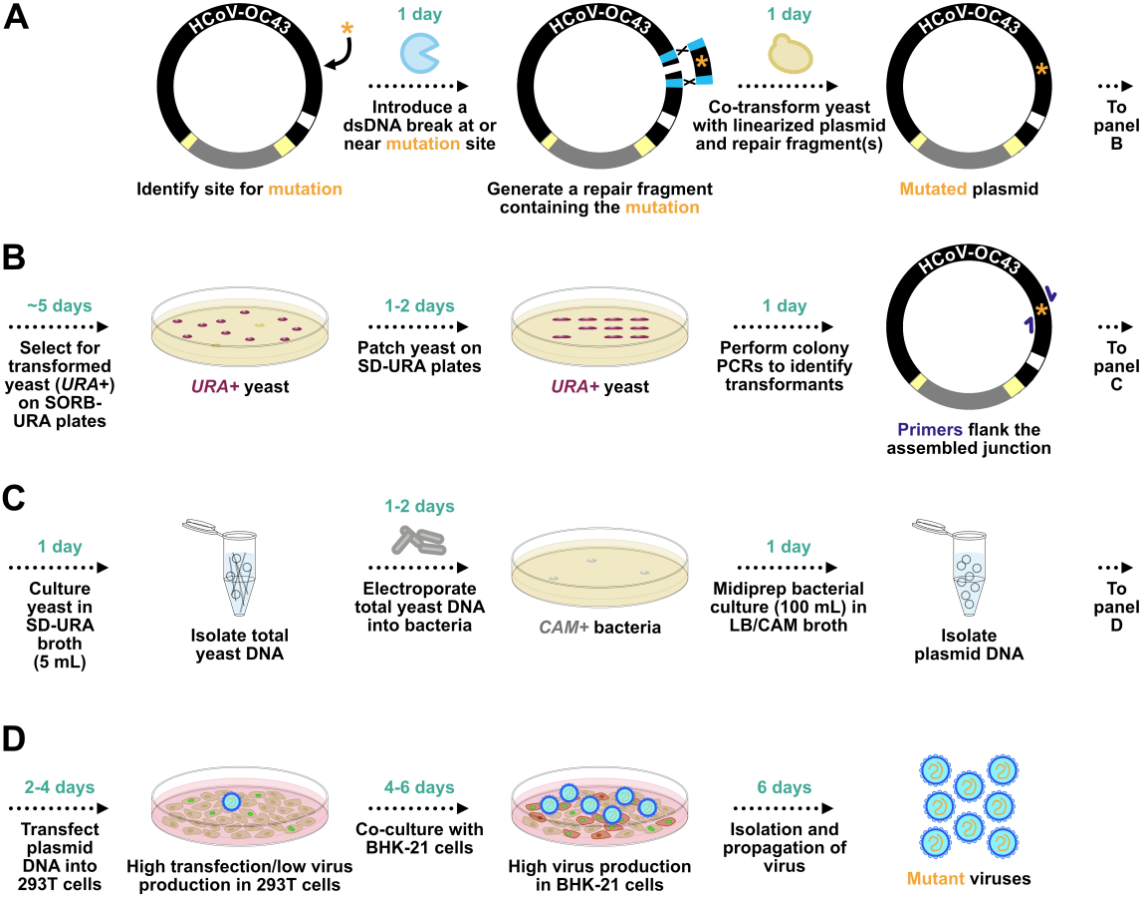
General overview of HCoV-OC43-YCpBAC mutagenesis, plasmid selection and propagation, and virus rescue. (A) Following the identification of a site (orange asterisk) for mutagenesis in silico, a DNA endonuclease is used to introduce a dsDNA break at or near the site for mutagenesis. A dsDNA homology-directed repair (HDR) fragment is designed carrying the mutagenized sequence with flanking homology regions (light blue rectangles) to sequences flanking the mutagenesis site in the HCoV-OC43-YCpBAC. The linearized HCoV-OC43-YCpBAC and the HDR fragment are co-transformed into yeast to create the mutated plasmid. (B) The yeast carrying the re-circularized HCoV-OC43-YCpBAC (maroon colonies) are selected on plates lacking uracil due to the *orotidine 5-phosphate decarboxylase (URA*) gene present in the YCpBAC. Yeast are patched onto SD-URA plates, and then colony PCRs are performed using primers (dark blue arrows) flanking the HDR fragment/YCpBAC junctions to identify correct transformants. (C) Yeast with the correctly mutated HCoV-OC43-YCpBAC are grown in liquid cultures lacking uracil, from which total yeast DNA is isolated and used to transform *Escherichia coli* with plasmid selection via chloramphenicol acetyltransferase (*CAM*) expression from the YCpBAC. (D) The mutated HCoV-OC43-YCpBAC is then transfected into 293T cells, followed by a co-culture with BHK-21 cells, to rescue mutant viruses.

**Figure 2.**
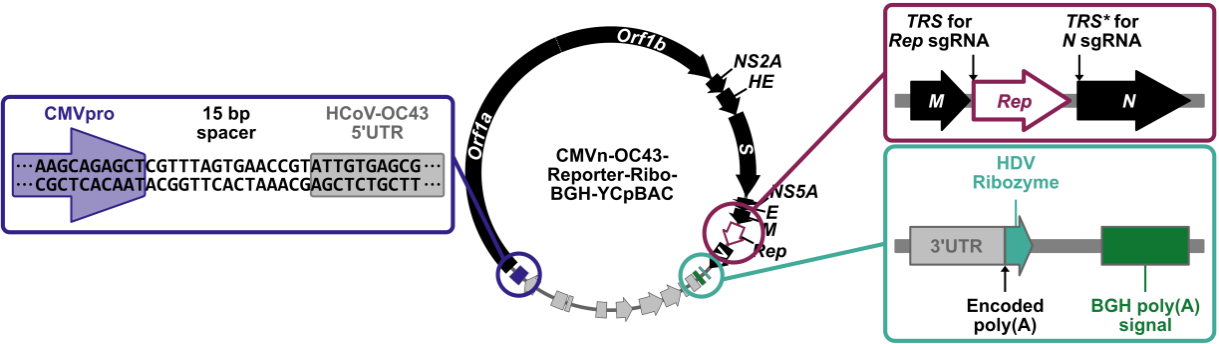
Transcription/RNA regulatory features of HCoV-OC43-YCpBAC plasmids to facilitate virus rescue in mammalian cells. The HCoV-OC43-YCpBACs described in this protocol are designed for virus rescue in mammalian cells. Upstream of the HCoV-OC43 5′ untranslated region (5′ UTR) sequence is a human cytomegalovirus promoter (CMVpro; blue highlighted region) sequence to initiate mRNA transcription. HCoV-OC43-YCpBACs reporter genes (*Rep*) are inserted in the intergenic region between the *M* and *N* genes under the control of the *N* transcription regulatory sequence (*TRS, TRS* for *Rep* sgRNA; maroon-highlighted region). The *N* gene in these reporter virus genomes is under the control of a duplicated version of the N TRS (TRS*; maroon-highlighted region). Immediately 3′ to the HCoV-OC43 3′ UTR sequence is an encoded poly(A) sequence followed by a hepatitis delta virus ribozyme (HDV ribozyme) sequence (teal-highlighted region) to generate mRNAs terminating in poly(A) sequences following transcription in mammalian cells. Downstream of the HDV ribozyme is a bovine growth hormone polyadenylation [BGH poly(A)] signal (teal-highlighted region) to facilitate mRNA transcription termination.

**Figure 3.**
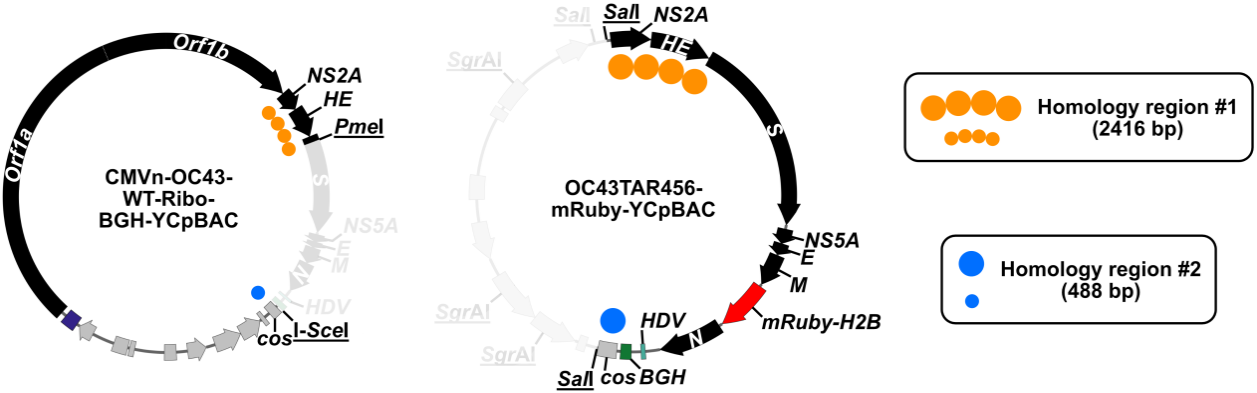
DNA fragments used in the generation of CMVn-OC43-mRuby-Ribo-BGH-YCpBAC. The CMVn-OC43-WT-Ribo-BGH-YCpBAC was digested with *Pme*I and I-*Sce*I to generate the YCpBAC fragment for TAR mutagenesis to insert the *mRuby-H2B* reporter gene into the OC43 genome. The HDR fragment carrying the reporter gene was generated from *Sal*I/*Sgr*AI digestion of OC43TAR456-mRuby-YCpBAC. The regions of homology to direct plasmid assembly are shown with orange circles (*NS2A-S*) and blue circles (*cos*). The greyed-out sections of the plasmids did not contribute to the assembly of CMVn-OC43-mRuby-Ribo-BGH-YCpBAC. All restriction endonuclease sites are underlined.

We used this yeast TAR approach to establish a system for assembly and mutagenesis of the human coronavirus OC43 (HCoV-OC43) genome, resulting in the generation of four independent full-length infectious clones of HCoV-OC43 in yeast centromeric plasmid/bacterial artificial chromosome vectors (HCoV-OC43-YCpBACs) [2]. This provides starting materials for others interested in rapid coronavirus genome assembly and mutagenesis to support research using HCoV-OC43. Our protocol describes the process of generating HCoV-OC43 mutant viruses from initial design to virus rescue (Figure 1) and is applicable to make mutant viruses in the wild-type or reporter virus backgrounds. These plasmids contain the required regulatory features to support the recovery of infectious viruses in mammalian cells (Figure 2). We provide an example of a two-fragment assembly to insert an *mRuby3-H2B* reporter gene into CMVn-OC43-WT-Ribo-BGH-YCpBAC (Figure 3); however, the same process applies for assemblies with more fragments using any of our HCoV-OC43-YCpBAC plasmids.

## Materials and reagents

**iological materials**

1. Yeast: *Saccharomyces cerevisiae* VL6-48N [24], genotype: *MATα, his3-Δ200, trpl-Δ1, ura3-Δ1, ade2-101, lys2, met14, cir°*), storage: -80 °C in 12.5% glycerol

2. Bacteria: Stbl3 *E. coli* (Thermo Fisher, catalog number: C737303), storage: -80 °C in 12.5% glycerol

3. Plasmids, HCoV-OC43-YCpBAC infectious clones (GenBank ID): CMVn-OC43-WT-Ribo-BGH-YCpBAC (PQ791637), CMVn-OC43-mClover-Ribo-BGH-YCpBAC (PQ791638), CMVn-OC43-mRuby-Ribo-BGH-YCpBAC (PQ791639), or CMVn-OC43-mCardinal-Ribo-BGH-YCpBAC (PQ791640), storage: 4 °C (short term) or -20 °C (long term) [2]

4. Plasmid, OC43-N: pGBW-m4134906 expressing HCoV-OC43-N (Addgene Plasmid #151960), storage: 4 °C (short term) or -20 °C (long term)

5. Optional plasmids, HCoV-OC43 genome fragments: OC43TAR1-YCpBAC (5′ UTR-*Nsp3*), OC43TAR2-YCpBAC (*Nsp3-Nsp11*), OC43TAR3-YCpBAC (*Nsp11-Nsp15*), OC43TAR4-YCpBAC (*NS2A-M*), OC43TAR6-YCpBAC (*N*-3′ UTR), OC43TAR123-YCpBAC (5′ UTR-*Orf1ab*), OC43TAR456-WT-YCpBAC (*NS2A*-3′ UTR), OC43TAR456-mClover-YCpBAC (*NS2A*-3′ UTR), OC43TAR456-mRuby-YCpBAC (*NS2A*-3′ UTR), and OC43TAR456-mCardinal-YCpBAC (*NS2A*-3′ UTR). Storage: 4 °C (short term) or -20 °C (long term) [2]

6. Virus: Betacoronavirus 1 strain OC43 (HCoV-OC43) (ATCC, catalog number: VR-1558), storage: -80 °C

7. Cell line: Human embryonic kidney (HEK) 293T cells (ATCC, catalog number: CRL-3216), storage: -196 °C (LN2)

8. Cell line: Human embryonic kidney (HEK) 293A cells (Thermo Fisher, catalog number: R70507), storage: -196 °C (LN2)

9. Cell line: Baby hamster kidney (BHK) 21 cells (ATCC, catalog number: CCL-10), storage: -196 °C (LN2)

10. Antibodies: Mouse anti-coronavirus, OC43 strain (OC43-N; Millipore, catalog number: MAB9012); rabbit anti-OC43-S (CUSABIO, CSB-PA336163EA01HIY); rabbit anti-OC43-HE (M. Desforges); rabbit anti-β-Actin (Cell Signaling, catalog number: 8457), goat anti-mouse IgG (H+L) cross-adsorbed secondary antibody, Alexa Fluor 555 (Invitrogen, catalog number: A21422); goat anti-rabbit IgG (H+L) cross-adsorbed secondary antibody, Alexa Fluor 647 (Invitrogen, catalog number: A21244); and goat anti-rabbit IgG (H+L) cross-adsorbed secondary antibody, Alexa Fluor 488 (Invitrogen, catalog number: A11008)

**Reagents**

1. Yeast extract (BioShop, catalog number: YEX401)

2. Peptone (Bacteriological; BioShop, catalog number: PEP403)

3. D-Glucose, anhydrous, reagent grade (BioShop, catalog number: GLU501)

4. Adenine hemisulfate salt (Sigma-Aldrich, catalog number: A9126)

5. Agar (Sigma-Aldrich, catalog number: A1296)

6. D-Sorbitol (Sigma-Aldrich, catalog number: S1876)

7. Ethylenediaminetetraacetic acid disodium salt dihydrate (EDTA) (Wisent, catalog number: 625-060 LG)

8. Sodium hydroxide (NaOH) (EMD, catalog number: SX0590-1)

9. Sodium phosphate dibasic heptahydrate (Sigma-Aldrich, catalog number: S9390)

10. Monosodium phosphate monohydrate (EMD, catalog number: SX0710-1)

11. Tris(hydroxymethyl)aminomethane (Sigma-Aldrich, catalog number: 154563)

12. Hydrochloric acid (HCl) (Fisher, catalog number: A144-500)

13. Zymolyase, 20T (MP Biomedicals, catalog number: 0832092), storage: -20 °C to -80 °C

14. Glycerol (Sigma-Aldrich, catalog number: G5516)

15. 14.3 M β-Mercaptoethanol (Fisher, catalog number: BP176-100), storage: 4 °C

16. Sodium dodecyl sulfate (SDS) (Wisent, catalog number: 800-100 LG)

17. Calcium chloride dihydrate (CaCl_2_) (Sigma-Aldrich, catalog number: C3881)

18. Poly(ethylene glycol), Avg 8,000 (Sigma-Aldrich, catalog number: P5413)

19. Yeast nitrogen base, w/o amino acids, w/ ammonium sulfate (BioShop, catalog number: YNB406)

20. Yeast synthetic drop-out medium supplement, without uracil (Sigma-Aldrich, catalog number: Y1501)

21. Glacial acetic acid (Fisher, catalog number: A38-500)

22. Ficoll, type 400 (Sigma-Aldrich, catalog number: F4375)

23. Bromophenol blue (BioShop, catalog number: BRO777)

24. Tryptone (bacteriological) (BioShop, catalog number: TRP402)

25. Sodium chloride (Fisher, catalog number: BP358-10)

26. Chloramphenicol (Sigma-Aldrich, catalog number: C0378)

27. 95% ethanol (Commercial Alcohols, catalog number: P016EA95)

28. Poly-L-lysine hydrochloride (Millipore-Sigma, catalog number: P2658), storage: -20 °C

29. Trypan blue solution, 0.4% (Thermo Fisher, catalog number: 15250061)

30. PEI MAX, transfection-grade linear polyethylenimine hydrochloride, MW 40,000 (Polysciences, catalog number: 24765)

31. Oligonucleotides for TAR hooks/YCpBAC amplification and diagnostic PCR primers, storage: -20 °C

*Note: Integrated DNA Technologies Ultramer DNA Oligos or PAGE purification of standard oligonucleotides is recommended for primers > 60 bp.*

32. KOD Xtreme Hot Start DNA polymerase (Millipore Sigma, catalog number: 71975-M), storage: -20 °C

33. UltraPure Salmon Sperm DNA solution (Thermo Fisher, catalog number: 15632011), storage: in aliquots at -20 °C

34. Monarch PCR & DNA Cleanup kit (New England Biolabs, catalog number: T1130)

35. Monarch Spin DNA Gel Extraction kit (New England Biolabs, catalog number: T1120)

36. *Taq* DNA Polymerase with ThermoPol buffer (New England Biolabs, catalog number: M0267L), storage: -20 °C

37. 10 mM deoxynucleotide (dNTP) solution mix (New England Biolabs, catalog number: N0447), storage: -20 °C

38. 100 bp DNA ladder (New England Biolabs, catalog number: N3231), storage: -20 °C

39. Gentra Puregene Yeast/Bact. kit (QIAGEN, catalog number: 158567)

*Note: This kit has been discontinued, but alternatives are available from other vendors (see Section H).*

40. QIAfilter Plasmid Midiprep kit (QIAGEN, catalog number: 12243)

41. Opti-MEM I Reduced Serum Medium (Thermo Fisher, catalog number: 31985070)

42. Dulbecco’s modified Eagle’s medium (DMEM; Thermo Fisher, catalog number: 11965118)

43. Heat-inactivated fetal bovine serum (FBS, Thermo Fisher, catalog number: A31607-01)

44. 100 U/mL penicillin, 100 μg/mL streptomycin, and 2 mM L-glutamine (Pen/Strep/Gln; Thermo Fisher, catalog numbers: 15140122 and 25030081)

45. RNeasy Plus Mini Kit (QIAGEN, catalog number: 74136)

46. Maxima H Minus First Strand cDNA Synthesis kit (Thermo Fisher, catalog number: K1652)

47. Luna Universal qPCR Master Mix (NEB, catalog number: M3003X)

48. DL-Dithiothreitol (Sigma-Aldrich, catalog number: D0632)

49. Trans-Blot Turbo RTA Midi 0.2 μm PVDF Transfer kit (Bio-Rad, catalog number: 1704273)

50. Bovine serum albumin (BioShop, catalog number: ALB001.500)

51. Tween-20 (Sigma-Aldrich, catalog number: P9416)

52. ssRNA ladder (NEB, catalog number: N0362S)

53. Streptavidin-HRP (Cell Signaling, catalog number: 3999)

54. Odyssey Blocking Buffer (LI-COR, catalog number: 927-50000)

55. Clarity Max Western ECL Substrate (Bio-Rad, catalog number: 1705062S)

**Solutions**

1. Yeast extract peptone dextrose (YEPD) medium (see Recipes)

2. YEPD/agar medium (see Recipes)

3. Sterile water (see Recipes)

4. 1 M Sorbitol solution (see Recipes)

5. 500 mM EDTA solution (see Recipes)

6. SPE solution (see Recipes)

7. 1 M Tris-HCl (pH 7.5) solution (see Recipes)

8. Zymolyase solution (see Recipes)

9. β-mercaptoethanol solution (see Recipes)

10. 2% SDS solution (see recipes)

11. 1 M CaCl_2_ solution (see Recipes)

12. STC solution (see Recipes)

13. PEG solution (see Recipes)

14. SOS medium (see Recipes)

15. SORB-URA medium (see Recipes)

16. SORB-URA/agar medium (see Recipes)

17. SORB-URA/agar overlay medium (see Recipes)

18. SD-URA medium (see Recipes)

19. SD-URA/agar medium (see Recipes)

20. CPZ solution (see Recipes)

21. 50× TAE buffer (see Recipes)

22. 6× DNA loading dye (see Recipes)

23. LB medium (see Recipes)

24. LB/agar medium with 17.5 μg/mL chloramphenicol for plates (see Recipes)

25. 35 mg/mL chloramphenicol (see Recipes)

26. Buffer EB solution (see Recipes)

27. 1 mg/mL Poly-L-lysine solution for coating tissue culture plastics (see Recipes)

28. 1 mg/mL PEI MAX solution (see Recipes)

29. 2× Laemmli buffer containing dithiothreitol/bromophenol blue (see Recipes)

**Recipes**

Note: Media and solutions with an asterisk (*) indicate those with known shelf lives that may need to be freshly prepared for optimal performance. Otherwise, refer to the expiry dates provided for individual reagents.

**1. YEPD medium**

**Table:** 

Reagent	Final concentration	Quantity or Volume
Yeast extract	1% w/v	5 g
Peptone	2% w/v	10 g
D-Glucose	2% w/v	10 g
Adenine hemisulfate	80 mg/L	40 mg
Type II water	n/a	Up to 500 mL

Autoclave for 15 min. Store at room temperature.

**2. YEPD/agar medium for plates**

**Table:** 

Reagent	Final concentration	Quantity or Volume
YEPD medium (Recipe 1)	n/a	250 mL
Agar	2% w/v	5 g

Autoclave for 15 min. Pour into Petri dishes. Store at 4 °C.

**3. Sterile water**

**Table:** 

Reagent	Final concentration	Quantity or Volume
Type II water	n/a	500 mL

Autoclave for 15 min. Store at room temperature.

**4. 1 M sorbitol solution**

**Table:** 

Reagent	Final concentration	Quantity or Volume
D-Sorbitol	1 M	91 g
Type II water	n/a	Up to 500 mL

Heat while stirring to dissolve. Autoclave for 15 min. Store at room temperature.

**5. 500 mM EDTA solution**

**Table:** 

Reagent	Final concentration	Quantity or Volume
EDTA disodium salt dihydrate	500 mM	18.61 g
Type II water	n/a	80 mL
Adjust to pH 8 with 5 N NaOH to dissolve the EDTA		
Type II water	n/a	Up to 100 mL

Store at room temperature.

**6. SPE solution**

**Table:** 

Reagent	Final concentration	Quantity or Volume
D-Sorbitol	1 M	91 g
Sodium phosphate dibasic heptahydrate	10 mM	1.04 g
Monosodium phosphate monohydrate	10 mM	0.16 g
500 mM EDTA solution (pH 8.0)	10 mM	10 mL
Type II water	n/a	Up to 500 mL

Heat while stirring to dissolve. Autoclave for 15 min. Store at room temperature.

**7. 1 M Tris-HCl (pH 7.5) solution**

**Table:** 

Reagent	Final concentration	Quantity or Volume
Tris(hydroxymethyl)aminomethane	1 M	60.55 g
Type II water	n/a	400 mL
Adjust to pH 7.5 with concentrated HCl.		
Type II water	n/a	Up to 500 mL

Store at room temperature.

**8. Zymolyase solution**

**Table:** 

Reagent	Final concentration	Quantity or Volume
Zymolyase, 20T	10 mg/mL	200 mg
1 M Tris-HCl (pH 7.5) solution	50 mM	1 mL
Type II water	n/a	14 mL
Stir until dissolved		
Glycerol	25% v/v	5 mL

Mix well and store in 500 μL aliquots at -20 °C.

9. β-mercaptoethanol solution*

**Table:** 

Reagent	Final concentration	Quantity or Volume
14.3 M β-Mercaptoethanol	14 mM	0.49 µL
Type II water	n/a	Up to 0.5 mL

Store in 100 μL aliquots at -20 °C (up to 6 months).

**10. 2% SDS solution**

**Table:** 

Reagent	Final concentration	Quantity or Volume
Sodium dodecyl sulfate	2% w/v	2 g
Type II water	n/a	Up to 100 mL

Store at room temperature.

11. 1 M CaCl_2_ solution*

**Table:** 

Reagent	Final concentration	Quantity or Volume
Calcium chloride dihydrate	1 M	7.35 g
Type II water	n/a	Up to 50 mL

Store at room temperature (up to 12 months).

**12. STC solution**

**Table:** 

Reagent	Final concentration	Quantity or Volume
D-Sorbitol	1 M	91 g
1 M Tris-HCl (pH 7.5) solution	10 mM	5 mL
1 M CaCl_2_ solution	10 mM	5 mL
Type II water	n/a	Up to 500 mL

Autoclave for 15 min. Store at room temperature.

13. PEG solution*

**Table:** 

Reagent	Final concentration	Quantity or Volume
Poly(ethylene glycol), Avg 8,000	20% w/v	5 g
1 M Tris-HCl (pH 7.5) solution	10 mM	0.25 mL
1 M CaCl_2_ solution	10 mM	0.25 mL
Type II water	n/a	Up to 25 mL

Heat while stirring to dissolve. Autoclave for 15 min or filter-sterilize. Store at room temperature.

*Note: Remake every 3 months to maintain high yeast transformation efficiency.*

**14. SOS medium**

**Table:** 

Reagent	Final concentration	Quantity or Volume
D-Sorbitol	1 M	18.2 g
1 M CaCl_2_ solution	6.5 mM	0.6 mL
Yeast extract	0.25% w/v	0.25 g
Peptone	0.5% w/v	0.5 g
Type II water	n/a	Up to 100 mL

Autoclave for 15 min. Store at room temperature.

**15. SORB-URA medium**

**Table:** 

Reagent	Final concentration	Quantity or Volume
D-Sorbitol	1 M	63.7 g
D-Glucose	2% w/v	7 g
Yeast nitrogen base, w/o amino acids, w/ ammonium sulfate	6.74 g/L	2.36 g
Yeast synthetic drop-out medium supplement, without uracil	1.92 g/L	0.67 g
Adenine hemisulfate	80 mg/L	28 mg
Type II water	n/a	300 mL
Heat while stirring to dissolve. Adjust to pH 5.6 if necessary		
Type II water	n/a	Up to 350 mL

Autoclave for 15 min. Store at room temperature.

**16. SORB-URA/agar medium for plates**

**Table:** 

Reagent	Final concentration	Quantity or Volume
SORB-URA medium (Recipe 15)	n/a	350 mL
Agar	2% w/v	7 g

Autoclave for 15 min. Pour into Petri dishes. Store at 4 °C.

**17. SORB-URA/agar overlay medium**

**Table:** 

Reagent	Final concentration	Quantity or Volume
SORB-URA medium (Recipe 15)	n/a	350 mL
Agar	2.5% w/v	8.75 g

Autoclave for 15 min. While still liquid, place 7 mL aliquots into 15 mL conical tubes and store at room temperature.

**18. SD-URA medium**

**Table:** 

Reagent	Final concentration	Quantity or Volume
D-Glucose	2% w/v	7 g
Yeast nitrogen base, w/o amino acids, w/ ammonium sulfate	6.74 g/L	2.36 g
Yeast synthetic drop-out medium supplement, without uracil	1.92 g/L	0.67 g
Adenine hemisulfate	80 mg/L	28 mg
Type II water	n/a	300 mL
Adjust to pH 5.6 if necessary		
Type II water	n/a	Up to 350 mL

Autoclave for 15 min. Store at room temperature.

**19. SD-URA/agar medium for plates**

**Table:** 

Reagent	Final concentration	Quantity or Volume
SD-URA medium (Recipe 18)	n/a	350 mL
Agar	2% w/v	7 g

Autoclave for 15 min. Pour into Petri dishes. Store at 4 °C.

**20. CPZ solution**

**Table:** 

Reagent	Final concentration	Quantity or Volume
Zymolyase solution (Recipe 8)	0.25 mg/mL zymolyase	0.5 mL
1 M Tris-HCl (pH 7.5) solution	50 mM	0.98 mL
Type II water	n/a	13.64 mL
Glycerol	25% v/v	4.88 mL

Mix well and store in 1 mL aliquots at -20 °C.

**21. 50× TAE buffer**

**Table:** 

Reagent	Final concentration	Quantity or Volume
EDTA disodium salt dihydrate	50 mM	93.05 g
Type II water	n/a	80 mL
Adjust to pH 8 with 5 N NaOH to dissolve the EDTA		
Type II water	n/a	600 mL
Tris(hydroxymethyl)aminomethane	2 M	242.2 g
Glacial acetic acid	1 M	57.1 mL
Type II water	n/a	Up to 1 L

Dilute 20 mL of 50× TAE with 920 mL of type II water before use. Store at room temperature.

**22. 6× DNA loading dye**

**Table:** 

Reagent	Final concentration	Quantity or Volume
Ficoll-400	15% w/v	3 g
500 mM EDTA solution (pH 8.0)	60 mM	2.4 mL
1 M Tris-HCl (pH 7.5) solution	20 mM	0.4 mL
2% SDS solution	0.48% v/v	4.8 mL
Bromophenol blue	0.03% w/v	6 μg
Type II water	n/a	Up to 20 mL

Mix well and store in 1 mL aliquots at -20 °C.

**23. LB medium**

**Table:** 

Reagent	Final concentration	Quantity or Volume
Tryptone	10 g/L	10 g
Yeast extract	5 g/L	5 g
Sodium chloride	10 g/L	10 g
Type II water	n/a	Up to 1 L

Autoclave for 15 min. Store at room temperature.

24. LB/agar medium with 17.5 μg/mL chloramphenicol for plates*

**Table:** 

Reagent	Final concentration	Quantity or Volume
LB medium (Recipe 23)	n/a	350 mL
Agar	2% w/v	7 g

Autoclave for 15 min. Once cool to touch, add 175 μL of 35 mg/mL chloramphenicol (Recipe 25). Pour into Petri dishes. Store at 4 °C (up to 6 months).

**25. 35 mg/mL chloramphenicol**

**Table:** 

Reagent	Final concentration	Quantity or Volume
Chloramphenicol	35 mg/mL	500 mg
95% ethanol	n/a	14.28 mL

Store in 0.5 mL aliquots at -20 °C.

**26. Buffer EB**

**Table:** 

Reagent	Final concentration	Quantity or Volume
1 M Tris-HCl (pH 7.5) solution	10 mM	0.5 mL
Type II water	n/a	49.5 mL

Store at room temperature.

**27. 1 mg/mL Poly-L-lysine solution for coating tissue culture plastics**

**Table:** 

Reagent	Final concentration	Quantity or Volume
Poly-L-lysine hydrochloride	1 mg/mL	50 mg
Type I water	n/a	50 mL

Filter sterilize (0.22 μm filter) and store at 4 °C.

28. 1 mg/mL PEI MAX solution*

**Table:** 

Reagent		Final concentration	Quantity or Volume	
PEI MAX, MW 40,000 (powder)		1 mg/mL	50 mg	
Type I water		n/a	35 mL	
Gradually adjust to pH 7.4 using diluted HCl/NaOH. Mix in a flask for 3–4 h at room temperature				
Readjust the pH to 7.4, if necessary				
Type I water	n/a			Up to 50 mL

Filter sterilize (0.22 μm filter) and store in 0.5 mL aliquots protected from light at 4 °C.

Transfection efficiency can be re-checked every 1–2 months by transfection of 293T cells with fluorescent protein encoding plasmids. Optimal transfection efficiencies should be > 80%.

**29. 2× Laemmli buffer containing dithiothreitol/bromophenol blue**

**Table:** 

Reagent		Final concentration	Quantity or Volume	
0.5 M Tris-Cl (pH 6.8)		125 mM	10 mL	
Sodium dodecyl sulfate		4% w/v	1.6 g	
Glycerol		20% v/v	8 mL	
Type II water	n/a			Up to 40 mL

Store at room temperature. When using to prepare lysates, add 100 μM dithiothreitol and 0.005% bromophenol blue directly to the lysate.

Laboratory supplies

1. Pipette tips (2 μL, 20 μL, 200 μL, 1,000 μL, non-filtered and filtered) (VWR)

2. Gel loading tips (200 μL) (Fisher, catalog number: 02-707-181)

3. Serological pipettes (5 mL, 10 mL, 25 mL) (Greiner Bio-One)

4. 1.5 mL tubes (Greiner Bio-One, catalog number: 616201)

5. 0.2 mL 8-strip PCR tubes (Frogga-Bio, catalog number: STF-A120)

6. Round-bottom culture tubes (VWR, catalog number: 60818-725)

7. Conical tubes (15 mL and 50 mL) (Greiner Bio-One)

8. Erlenmeyer flasks (50 mL, 250 mL, 500 mL)

9. Petri dishes (VWR, catalog number: 25384-088)

10. Electroporation cuvettes, 0.2 cm gap width (VWR, catalog number: 89047-208)

11. 0.2 μm PES syringe filters (Sarstedt, catalog number: 83.1826.001)

12. 50 mL syringes (BD, catalog number: 309653)

13. 96-well assay plates, F-bottom and U-bottom (Greiner Bio-One, catalog numbers: 655101 and 650101)

14. Hard-shell 96-well PCR plates, low profile, thin wall, skirted, white/clear (Bio-Rad, catalog number: HSP9601)

15. Microseal 'B' PCR plate sealing film, adhesive, optical (Bio-Rad, catalog number: MSB1001)

16. Reagent reservoirs (100 mL) (VWR, catalog number: 89094-656)

17. Tissue culture-treated plates, dishes, and flasks (Greiner Bio-One)

## Equipment

1. Pipettes (single-channel, 0.2–2 μL, 2–20 μL, 20–200 μL, 200–1,000 μL; 8 or 12-channel, 1–10 μL, 20–200 μL) (Gilson)

2. Pipette filler (Thermo Fisher, model: S1)

3. Mastercycler Pro S (96-well format) thermal cycler (Eppendorf, model: 6325)

4. Wide Mini-Sub Cell GT Horizontal Electrophoresis System, 15 × 10 cm tray (Bio-Rad, catalog number: 1704469)

5. 26-well comb × 2, multichannel pipette compatible (Bio-Rad, catalog number: 1704457)

6. PowerPac Basic Power Supply (Bio-Rad, catalog number: 1645050)

7. ChemiDoc MP Imaging System with Blot/UV/stain-free sample tray (Bio-Rad, catalog number: 12003154/12003028)

8. Kelvin^+^ incubator shaker (Kuhner, models: SMZ1220 and SMZ1200)

*Note: Incubator needs to be able to operate at 30 °C with/without shaking.*

9. Inverted-phase contrast microscope with 20× objective (Olympus, CKX41 or Nikon, Diaphot or similar)

10. Hemocytometer (Marienfeld, catalog number: 0640010)

11. Refrigerated benchtop centrifuge with buckets for 50/15 mL tubes and 96-well plates (Eppendorf, model: 5810R)

12. Microcentrifuge (Eppendorf, model: 5425)

13. Refrigerated microfuge (Eppendorf, model: 5415R)

14. Water bath (Fisher Scientific, model: Isotemp 220) (capable of 37–60 °C)

15. Gene Pulser II Electroporation System with Pulse Controller PLUS module (Bio-Rad, model: 340BR)

16. Class II biosafety cabinet (Baker, model: SG403A, Class II, Type A2)

17. CO_2_ incubator for cell culture (37 °C) (Sanyo, model: MCO18-AIC)

18. Trans-Blot Turbo Transfer System (Bio-Rad, catalog number: 1704150)

19. Spectrolinker UV Crosslinker (Spectronics, model: XL-1000)

## Software and datasets

1. SnapGene (Dotmatics, v 8.0.3, used under license agreement) or similar molecular biology software for visualizing and manipulating DNA sequences in silico

## Procedure

A. Considerations for HCoV-OC43-YCpBAC TAR mutagenesis strategies

To perform mutagenesis of HCoV-OC43-YCpBAC plasmids (WT, mClover, mRuby, or mCardinal), dsDNA fragments containing mutations must have two ~50 bp regions of homology (known as “TAR hooks”) with the HCoV-OC43-YCpBAC at or near the mutation site to drive recombination-mediated assembly of a mutagenized plasmid (Figure 1A). When a desired location for mutagenesis is selected, the first important consideration is the location and method for introducing a dsDNA break (DSB) at or near the site of a mutation. An easy method to introduce DSBs in HCoV-OC43-YCpBACs is to use restriction endonucleases (Figure 1A). While the number and location of unique cutting or double-cutting enzymes are limited, there are advantageous restriction sites dispersed throughout the HCoV-OC43 genome, within the *mClover3-H2B, mRuby3-H2B*, and *mCardinal* reporter genes, as well as in the YCpBAC or transcription/RNA regulatory sequences that can be used to mutagenize these plasmids (Table 1). It is also possible to introduce silent mutations within a viral ORF to create a novel, unique restriction site to facilitate the generation of multiple mutant sequences in the same gene. However, it is worth considering that changes to the viral RNA sequence with silent mutations may have additional impacts on RNA secondary structures that can affect viral replication. Additional approaches to generating single dsDNA breaks within HCoV-OC43-YCpBACs in vitro include *Thermus thermophilus* DNA-guided Argonaute (TtAgo) cleavage [25] or Cas9 RNA-guided cleavage [26]. To insert a dsDNA break using TtAgo, two phosphorylated guide DNAs are used to target TtAgo to both strands of the plasmid at the desired location, introducing two nicks resulting in a dsDNA break. For Cas9 cleavage, a synthetic single-guide RNA for Cas9 (Cas9-sgRNA) can be purchased or generated using in vitro transcription with T7 RNA polymerase from partially complementary DNA oligonucleotides. The Cas9-sgRNA can then direct the Cas9 enzyme to introduce a DSB at the precise location of interest. Both TtAgo and Cas9 nuclease can be purchased from New England Biolabs (NEB).

A second important consideration is the design of the dsDNA fragment(s) that will simultaneously repair the DSB and mutagenize the plasmid. These dsDNA fragments should be designed to flank the mutation site with corresponding homologous sequences 5′ and 3′ of the substitution, insertion, or deletion to mediate homology-directed repair (HDR) in yeast (Figure 1A). While the best HDR fragments begin and end with sequences that are identical to the sequences flanking the mutation site, there is some tolerance for mismatches at the extreme 5′ and 3′ ends of the DNA fragment (i.e., restriction site overhangs) or unequal TAR hook length. These dsDNA fragments can be generated using PCR amplification, restriction digestion, or custom DNA synthesis. With TAR assembly, multiple mutations/HDRs can be performed at different locations in the plasmid in the same reaction, provided that overlapping dsDNA fragments to facilitate the assembly are present. Other coronavirus reverse genetics systems, such as circular polymerase extension reaction-based methods, rely on PCR-amplified dsDNA fragments of the entire viral genome and vector sequences for assembly [27–31]. Thus, PCR amplifying the HCoV-OC43-YCpBAC in multiple, overlapping dsDNA fragments would also be an acceptable TAR assembly strategy. Our initial capture of HCoV-OC43 dsDNA fragments required PCR amplification of the 10-kbp YCpBAC plasmid as well as reverse transcription and PCR of large sections of HCoV-OC43 cDNA [2], demonstrating that the KOD Xtreme Hot Start DNA polymerase performs well in long-range PCR amplification and is suitable for amplifying large dsDNA fragments for assembly. However, unwanted errors can arise through PCR, and the probability of these errors increases when greater proportions of the viral genome are amplified by PCR for assembly [32].

Our HCoV-OC43 YCpBACs were designed to allow for virus rescue directly from assembled plasmids using transfected mammalian cells. This plasmid DNA-launched rescue technique has been used by multiple groups to rescue infectious coronaviruses [4,7,9,11,12,15,16,19,27,28,30,33–37], including HCoV-OC43 [2,10,31]. Our reverse genetics system uses a plasmid-based rescue system originally developed for Sindbis virus [38]. A CMV promoter (CMVpro) proximal to the HCoV-OC43 5′ UTR sequence is used to initiate mRNA synthesis in transfected cells (Figure 2, highlighted in blue). Transcription continues through an encoded poly(A) tail and a sequence encoding the hepatitis delta virus (HDV) ribozyme and terminates at a bovine growth hormone (BGH) polyadenylation signal sequence (Figure 2, highlighted in cyan). The HDV ribozyme cleaves the transcript immediately following the encoded poly(A) sequence to generate a viral genome-like capped and polyadenylated mRNA. Our reporter viruses utilize reporter genes (*Rep*) not fused to or inserted in place of any viral genes. These *Rep* genes are instead encoded by stand-alone sgRNAs, driven by the authentic *N* transcription regulatory sequence (*TRS*), where *N* sgRNA expression is maintained by the insertion of an additional copy of the N TRS (TRS*) (Figure 2, highlighted in maroon). While these reporter viruses and a yeast-assembled, wild-type HCoV-OC43 (OC43^YA^) were rescued using a 293T/BHK21 co-culture system (Figure 1D and [2]), it is important to note that some viral mutants will be more difficult to rescue due to growth defects. In these instances, *trans* complementation by co-transfection of the gene of interest or using stable cell lines expressing the protein of interest may mitigate the effects of some growth defects during viral rescue or while generating viral stocks. Using a HCoV-OC43-YCpBAC expressing a reporter protein as a backbone for mutagenesis will facilitate detecting the low-frequency viral production during the initial stages of HCoV-OC43 rescue.

**Table 1.** Useful restriction endonucleases for HCoV-OC43 plasmid mutagenesis. Listed are unique or multi-cutting restriction endonuclease sites that facilitate mutagenesis of the indicated ORFs. Cut sites do not need to be precisely at the location for mutagenesis.

ORF or feature	Restriction endonuclease (site)	# Cut sites, cut locations	Plasmids*
5′ UTR/*Nsp1*	*Avr*II (5′CCTAGG)	2, 5′ UTR + *NS2A*	All
*Nsp5*	*Bsi*WI (5′CGTACG)	1	WT, mClo, mCard
*Nsp12/RdRp*	*Fsp*AI (5′RTGCGCAY)	2, *Nsp12* + YCpBAC	All
*Nsp13*	*Xho*I (5′CTCGAG)	2, *Nsp13* + YCpBAC	WT, mClo, mRuby
*Nsp13*	*Psp*XI (5′VCTCGAGB)	2, *Nsp13* + YCpBAC	All
*Nsp14*	*Mlu*I (5′ACGCGT)	2, *Nsp14* + *Nsp14*	All
*Nsp14*	*Kas*I (5′GGCGCC)	1	All
*Nsp14*	*Cla*I (5′ATCGAT)	1	All
*NS2A*	*Avr*II (5′CCTAGG)	2, 5′ UTR + *NS2A*	All
*S*	*Pme*I (5′GTTTAAAC)	1	All
*mRuby*	*Bsi*WI (5′CGTACG)	2, 5′ UTR + *mRuby*	mRuby
*Reporter genes*	*Bam*HI (5′GGATCC)	2, *Reporter* + YCpBAC	mClo, mRuby, mCard
*N*	*Kfl*I (5′GGGWCCC)	1	All
*N*	*Bbv*CI (5′CCTCAGC)	1	All
3′ UTR	*Bst*EII (5′GGTNACC)	2, 3′ UTR + YCpBAC	All
HDV Ribozyme	*Cpo*I (5′CGGWCCG)	1	All
YCpBAC	*Bam*HI (5′GGATCC)	1 (3′ of HDV Ribozyme)	WT
YCpBAC	*Pac*I (5′TTAATTAA)	1 (5′ of BGH poly(A))	All
YCpBAC	I-*Sce*I (5′TAGGGATAACAGGGTAAT)	1 (3′ of BGH poly(A))	All
YCpBAC	*Sgr*AI (5′CRCCGGYG)	3, YCpBAC	All
YCpBAC	*Sma*I (5′CCCGGG)	1	WT, mClo, mRuby
YCpBAC	*Mre*I (5′CGCCGGCG)	1	All

Abbreviations: BGH, bovine growth hormone; HDV, hepatitis delta virus; mCard, mCardinal; mClo, mClover; N, nucleocapsid; NS, non-structural; Nsp, non-structural protein; S, spike; UTR, HCoV-OC43 untranslated region; WT, wild type; YCpBAC, yeast centromeric plasmid/bacterial artificial chromosome. * “All” indicates that these restriction sites are present in the WT, mClover, mRuby, and mCardinal HCoV-OC43-YCpBACs.

B. Streaking of VL6-48N yeast (Day 1)

1. Streak a glycerol stock of yeast VL6-48N onto a YEPD/agar plate and incubate overnight at 30 °C.

C. Preparation of DNA fragments and yeast cultures for TAR mutagenesis (Day 2)

1. Introduce a dsDNA break at or near the site to be mutagenized in the HCoV-OC43-YCpBAC using an endonuclease. Digest > 1 μg of plasmid to ensure there is enough for multiple TAR reactions or controls (see General notes 1 and 2). Figure 3 provides an example of the YCpBAC fragment (*Pme*I/I-*Sce*I-digested CMVn-OC43-WT-Ribo-BGH-YCpBAC) used to generate CMVn-OC43-mRuby-Ribo-BGH-YCpBAC.

2. Generate an HDR fragment by PCR or restriction digestion of an existing mutagenized sequence. Ensure that the HDR fragment contains ~50 bp of sequence at both the 5′ and 3′ ends that are homologous to sequences flanking the mutation in the HCoV-OC43-YCpBAC to direct the recombination reaction. Figure 3 provides an example of the HDR fragment (*Sal*I/*Sgr*AI-digested OC43TAR456-mRuby-YCpBAC) used to generate CMVn-OC43-mRuby-Ribo-BGH-YCpBAC.

*Note: In this example, SgrAI digestion of OC43TAR456-mRubyH2B-Ribo-BGH-YCpBAC is used to disrupt the YCpBAC sequence in this plasmid to reduce background transformants after plating the TAR reactions (step E5). Including this restriction endonuclease eliminates the need to gel extract the desired HDR fragment from the vector backbone.*

3. Determine the concentration (ng/μL), length (bp), and number of molecules (pmol) of each DNA fragment (see General note 3). Calculate the amounts of each DNA fragment needed for all transformations. See Table 2 for example calculations for an assembly reaction containing a 15:1 ratio of pmol mutagenesis fragments to pmol YCpBAC fragments.

4. Combine all DNA fragments together. Use ~100 ng of the YCpBAC-containing fragment and ≥ 5-fold molar excess of non-YCpBAC fragment(s) (see General note 4).

**Critical:** Using YCpBAC fragment amounts greater than 200 ng may lead to a high level of background colonies on the SORB-URA plates after transformation. Prepare a “vector only” control TAR reaction with an equal amount of YCpBAC to the assembly reactions to monitor the level of background transformants.

*Notes:*

*1. When performing TAR fragment capture using PCR-amplified YCpBACs with TAR hooks, reduce the YCpBAC fragment amount to ~10 ng.*

*2. Keep the total volume of the solution of DNA fragments to 50 μL or less.*

**Table 2.** Example calculations for the CMVn-OC43-mRuby-Ribo-BGH-YCpBAC assembly reaction (Figure 3)

Fragment name	μg†	V (μL)	[ng/μL]	Size (bp)	pmol/μL‡	pmol	V in assembly (μL)
CMVn-OC43-WT-Ribo-BGH-YCpBAC +*Pme*I/I-*Sce*I	1.64	20	82	33734	0.0037	0.02	5.41 μL (444 ng)
OC43TAR456-mRubyH2B-Ribo-BGH-YCpBAC +*Sal*I/*Sgr*AI	1.13	20	56	11595	0.0415	0.3	7.23 μL (405 ng)

† $\;\mu g\;of\;fragment\;in\;the\;restriction\;digest=\left(Total\;\mu g\;DNA\times \frac{Fragment\;size\;(bp)}{Total\;plasmid\;size\;(bp)}\right)$

‡ $pmol/\mu L\;=\left(\mu g\;DNA\times 10^{6}\frac{pg}{\mu g}÷660\frac{pg}{pmol}÷\#of\;nucleotides\right)÷\mu L$

Abbreviations: bp, base pairs; V, volume.

5. Store the DNA at -20 °C.

**Pause point:** DNA can be stored at -20 °C until ready to perform the subsequent yeast culture, spheroplasting, and transformation steps.

6. Start 2–3 independent overnight cultures of yeast in 50 mL of YEPD medium from small-to-medium-sized isolated colonies. Incubate at 30 °C overnight with vigorous shaking (120 rpm, Kelvin+ incubator shaker). Using multiple small-to-medium colonies allows for one or more overnight cultures to reach the desired cell density by the following morning, without the need for subsequent incubations to reach log phase growth.

*Note: 10 mL cultures can be inoculated and grown overnight, but these cultures will need to be diluted and subsequently grown to log phase the following day.*

D. Yeast spheroplast preparation (Day 3)

**Figure 4.**
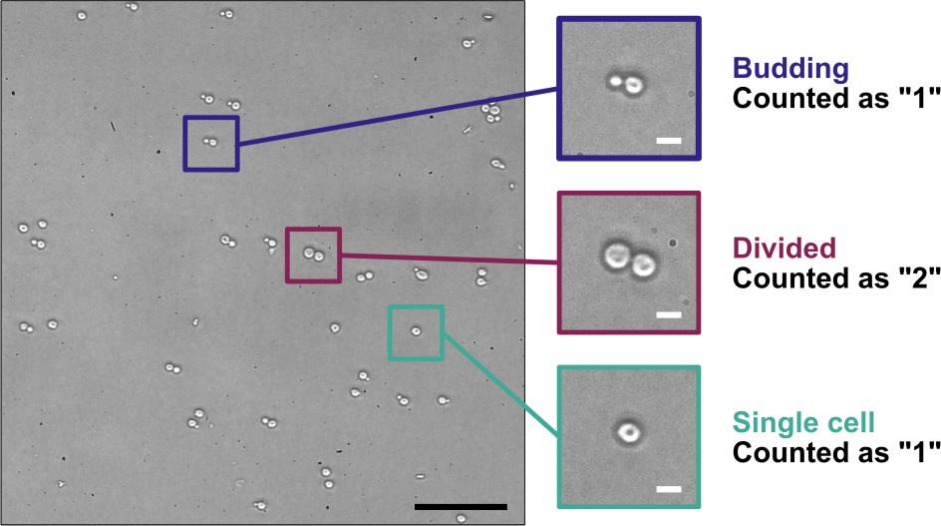
Counting yeast cells using a hemocytometer. Yeast in a log-phase culture were spotted onto a microscope slide with a cover glass and imaged with a Nikon Diaphot inverted-phase contrast microscope using a 20× objective (PhL filter) fitted with a Nikon D300s digital camera. The three areas of magnification illustrate yeast cells undergoing budding (blue-highlighted region), yeast cells that have divided (maroon-highlighted region), and a single yeast cell (teal-highlighted region), and how those different stages are counted for determining yeast cell concentration. Scale bars = 50 μm (black) or 5 μm (white).

1. Dilute 50 μL of yeast culture with 450 μL of sterile water and measure the density of each 1/10 diluted yeast culture using a hemocytometer (Figure 4). When counting yeast with a phase-contrast microscope, count all single cells and budding cells as “1” and any divided cells as “2”. The desired endpoint is 2–5 × 10^7^ cells/mL.

*Notes:*

*1. Seeing many divided cells in groups or two or four is a good sign that the culture is in log phase.*

*2. If the cultures are < 2 × 10^7^ cells/mL, they can be left to grow longer at 30 °C with vigorous shaking.*

*3. If the cultures are > 5 × 10^7^ cells/mL, select one culture and dilute to 5 × 10^6^ cells/mL in 50 mL (final) of YEPD medium and incubate at 30 °C with vigorous shaking until the endpoint is reached (~4 h). This step will also apply for 10 mL overnight cultures (see step C6).*

*4. Yeast culture density can also be assessed by measuring OD_600_. It will be necessary to determine the OD_600_ value corresponding to various yeast cell densities for each spectrophotometer.*

2. Pellet the yeast cells at 3,000× *g* for 5 min at 4 °C in a 50 mL conical tube. Discard the supernatant.

3. Set a water bath to 55 °C and warm the appropriate number of tubes of SORB-URA/agar overlay medium. The number of tubes will correspond to the number of spheroplast transformations being performed.

4. Add sterile water up to the 50 mL mark (~50 mL) and resuspend the pellet with vortexing. Pellet the yeast cells at 3,000× *g* for 5 min at 4 °C and discard the supernatant.

5. Add 1 M sorbitol solution up to the 50 mL mark (~50 mL) and resuspend the pellet with vortexing. Pellet the yeast cells at 3,000× *g* for 5 min at 4 °C and discard the supernatant.

**Pause point:** Prior to centrifugation, yeast cells in 1 M sorbitol solution can be stored overnight at 4 °C.

6. Add 20 mL of SPE solution to the yeast cell pellet and resuspend with vortexing. Add 160 μL of zymolyase solution (10 mg/mL) and 40 μL of β-mercaptoethanol solution, mix well, and then incubate at 30 °C with gentle shaking (40 rpm, Kelvin^+^ shaker) for 20 min.

**Critical:** Using 160 μL of 10 mg/mL zymolyase solution was empirically determined to result in near complete conversion to spheroplasts in a 20–30 min time frame. Varying the volume of zymolyase solution used may be needed to achieve optimal results within 30 min (see General note 5).

7. During the zymolyase treatment, check the tubes of SORB-URA/agar overlay medium from step D3 to see if they have melted. Vortex to mix. If the overlay medium has not yet melted, microwave the tubes upright in a beaker with the caps loosened in 15-s intervals with vortexing in between. Once the overlay solutions are melted, return them to the 55 °C water bath to cool down to 55 °C prior to use. Also, bring SORB-URA/agar plates to room temperature if previously stored at 4 °C.

**Caution:** The overlay solution in the 15 mL tubes will boil over if it is heated for too long. Use caution when handling and vortexing the tubes, as the solution will be hot.

**Figure 5.**
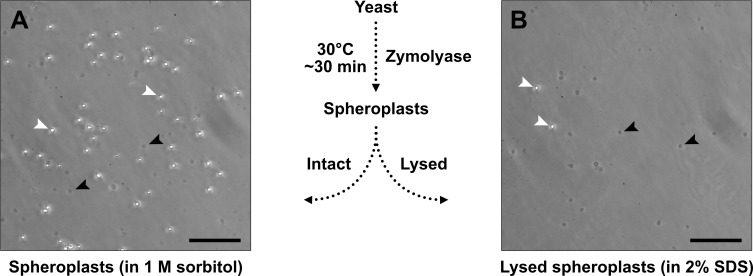
Assessing yeast spheroplast formation using a hemocytometer. Yeast spheroplasts were diluted in 1 M sorbitol (A) or 2% sodium dodecyl sulfate (SDS) (B), spotted onto microscope slides with a cover glass, and imaged with a Nikon Diaphot inverted-phase contrast microscope using a 20× objective (Ph2 filter) fitted with a Nikon D300s digital camera. Spheroplasts in 1 M sorbitol (A) will help to determine spheroplast integrity, and those in 2% SDS (B) will help to determine how complete spheroplast formation is. Intact yeast or intact spheroplasts appear bright (white arrowheads), whereas lysed or damaged spheroplasts appear dark grey (black arrowhead). Arrowheads provided for illustration, not quantitation. Scale bars = 50 μm.

8. Check the level of spheroplasting using a hemocytometer. Prepare two 1.5 mL tubes of spheroplasts, each with 10 μL of spheroplasts in SPE/β-mercaptoethanol/zymolyase from step D6. To one tube, add 90 μL of 1 M sorbitol and gently mix. To the second tube, add 90 μL of 2% SDS solution and gently mix. View each sample in separate chambers of the hemocytometer using a phase-contrast microscope. Intact spheroplasts (in 1 M sorbitol) should appear round and bright (Figure 5A, white arrowheads), similar to untreated yeast cells. Spheroplasts lysed by incubation with 2% SDS should appear grey and physically distinct from intact cells (Figure 5B, black arrowheads). It is normal to observe some spheroplast lysis even in 1 M sorbitol (Figure 5A, black arrowheads) due to their fragility.

**Critical:** When nearly all the spheroplasts in the 2% SDS solution appear gray/lysed, proceed to step D9. Incubate longer at 30 °C if spheroplasting is not complete and check again in 5-min intervals.

**Critical:** Spheroplasts are fragile. Avoid vortexing or vigorous pipetting.

9. Add 1 M sorbitol solution up to the 50 mL mark (~30 mL) to dilute the zymolyase. Pellet the spheroplasts at 700× *g* for 10 min at 4 °C and discard the supernatant.

**Critical:** Pellet the spheroplasts immediately once the zymolyase incubation is complete.

10. Add 1 M sorbitol solution up to the 50 mL mark (~50 mL) and resuspend the spheroplasts with gentle rocking or pipetting. Pellet the spheroplasts at 700× *g* for 10 min at 4 °C and discard the supernatant.

11. Repeat step D10 to perform an additional wash of the spheroplasts using ~50 mL of 1 M sorbitol. Discard the supernatant.

12. Resuspend the spheroplasts in 2 mL of STC solution. This suspension of spheroplasts is enough to perform ten transformations.

**Pause point:** Spheroplasts are stable for up to 1 h at room temperature.

**E. Spheroplast transformations (Day 3)**

1. In a 1.5 mL tube, add 200 μL of prepared spheroplasts (from step D12) with the DNA solution containing the YCpBAC fragment and the mutagenesis fragment(s) prepared in step C4. Also, prepare a corresponding “vector only” transformation using 200 μL of prepared spheroplasts (from step D12) and only the YCpBAC fragment in a separate tube. Incubate at room temperature for 10 min.

**Optional:** Add denatured salmon sperm DNA as a carrier to bring the total DNA amount up to 5 μg (see General note 6).

2. Add 900 μL of PEG solution into each 1.5 mL tube and mix gently by inversion. Incubate at room temperature for 20 min.

3. Pellet the spheroplasts at 700× *g* for 5 min in a microfuge and gently aspirate off the supernatant.

*Note: The spheroplasts will pellet against the side and at the bottom of the 1.5 mL tube. Care is needed to remove the supernatant without disturbing the spheroplasts.*

4. Add 800 μL of SOS solution and gently pipette to resuspend the spheroplasts. Incubate at 30 °C for 30 min without shaking.

5. Gently pipette the spheroplasts in SOS solution to resuspend and transfer into a 15 mL tube with melted SORB-URA/agar overlay medium. Gently mix by inverting the tube twice and immediately pour onto SORB-URA/agar plates.

**Critical:** Waiting too long to pour the spheroplasts in the warmed overlay medium onto the SORB-URA/agar plates will result in the agar solidifying in the 15 mL tube.

*Note: The SORB-URA/agar overlay medium is used to protect the transformed spheroplasts from osmotic stress until they regenerate their cell walls after plating.*

6. Incubate the plates at 30 °C for 4–6 days until colonies appear. Alternatively, plates can be left at room temperature, but colonies will be slower to appear.

*Note: The “vector only” transformation helps determine the amount of background transformants for a given YCpBAC fragment. A successful assembly reaction should yield more transformants than the “vector only” transformation.*

**F. Patching yeast transformants onto SD-URA plates (~Day 8)**

1. Patch at least 23 yeast colonies per transformation (step E1) onto SD-URA/agar plates and number to identify clones (Figure 1B). Incubate the plates at 30 °C for 1–2 days.

*Notes:*

*1. The yeast will grow on top of or within the SORB-URA/agar overlay. These can be picked with sterile tips or toothpicks.*

*2. It is not necessary to patch yeast colonies from the “vector only” transformations. These plates are for visual examination of the level of background transformants only.*

G. Screening yeast transformants by yeast colony PCR (~Day 9)

1. Thaw all reagents for colony PCRs on ice, including CPZ solution, ThermoPol buffer, dNTP solution, and 100 μM primers.

2. Add 20 μL of CPZ solution per well of a 96-well PCR plate. Use 24 wells per transformation reaction to screen 24 independent clones (see General note 5). Store the plate on ice.

*Note: Provided the TAR mutagenesis design is correct and the transformations are successful, the frequency of correct recombinants should exceed 20%. Not all 24 clones may need to be screened by colony PCR, but it is worthwhile preparing 24 lysates at this stage to have additional samples to screen later, if required.*

3. Take a small but visible amount of patched yeast from the SD-URA/agar plate and transfer to the 96-well PCR plate. The solution should be lightly turbid. Repeat for all clones to screen.

*Note: Adding too much yeast to the CPZ solution may hinder lysis and subsequent PCR target amplification.*

4. Seal the plate and incubate at 37 °C for 20 min in a 96-well thermal cycler to lyse the yeast. Store the plate on ice when complete.

**Pause point:** This yeast lysate plate can be sealed and stored at -20 °C.

5. Prepare PCR master mixes with primers suitable to screen for the inserted mutation(s). Each 25 μL PCR will contain the following components (volumes per reaction): 2.5 μL of 10× ThermoPol Buffer, 0.75 μL of 10 mM dNTPs, 0.125 μL of 100 μM forward primer, 0.125 μL of 100 μM reverse primer, 0.125 μL of *Taq* polymerase, and 18.875 μL of nuclease-free water.

*Note: If more than one diagnostic PCR is required, multiplex PCR can be performed, provided the diagnostic primer pairs are designed to yield different amplicon sizes. Alternatively, sequential PCRs can be performed.*

6. In a new 96-well plate stored on ice, add 22.5 μL of PCR master mix per well and 2.5 μL of yeast lysate from step G4. Screen at least 11 yeast lysates by colony PCR on the first attempt, with the option to screen additional lysates in subsequent PCRs. Include a negative control PCR for each master mix used that contains 2.5 μL of CPZ solution.

*Note: This step can be expedited using multi-channel pipettes.*

7. Seal the plate and cycle the PCRs using the following conditions in a 96-well thermal cycler: 95 °C for 3 min × 1 cycle, [95 °C for 15 s, 50–68 °C (see note below) for 20 s, 68 °C for 1 min per kb] × 35 cycles, 68 °C for 3 min × 1 cycle, 10 °C hold.

*Note: Primers should be designed to have an annealing temperature of 50–68 °C. The annealing temperature for each primer pair can be estimated with the New England Biolabs Tm Calculator (tmcalculator.neb.com) using the following options: Taq DNA Polymerase, Taq DNA Polymerase with ThermoPol Buffer, 500 nM primer concentration. Primers should be designed to yield amplicons of 100–1,500 bp in size. Larger amplicons will be more difficult to amplify by colony PCR.*

**Pause point:** The 96-well plate of colony PCRs can be stored at -20 °C.

8. While the PCRs are running, pour a 1.8% agarose gel in 1× TAE buffer in a wide mini-sub cell casting tray with 2 × 26-well combs. Do not add ethidium bromide.

*Notes:*

*1. 1.8% agarose gels can be used to visualize colony PCR amplicons of 100–1,500 bp in length. Larger amplicons will require a lower percentage of agarose in the gel to resolve properly.*

*2. Since ethidium bromide migrates in the opposite direction compared to DNA, when using two 26-well combs placed at the top and middle of the casting tray, it is important to stain the gel after running to visualize DNA in the bottom half of the gel.*

9. To each PCR, add 5 μL of 6× DNA loading dye.

10. Place the agarose gel in the wide mini-sub cell apparatus and add enough 1× TAE to cover the gel. Load the wells with 15 μL of PCR+loading dye or 5 μL per lane of 100 bp DNA ladder. Electrophorese the gel at 85 V (constant) for 45 min using a PowerPac Basic Power Supply (or similar).

**Critical:** When running samples in the wells closest to the anode, monitor the dry front to ensure it does not run out of the gel. This front will be approximately equivalent to DNA ~100 bp in size.

*Note: Loading gels with 26-well combs can be expedited using multi-channel pipettes.*

11. Stain the gel following electrophoresis in a solution containing 1× TAE with 0.5 μg/mL ethidium bromide. Incubate the gel with gentle rocking at room temperature for 20 min.

12. Image the gel using a ChemiDoc MP Imaging System with Blot/UV/stain-free sample tray (or similar).

13. Identify positive transformants. If additional diagnostic PCRs are required for the same clones, repeat steps G5–G12 with the additional primer sets using the same yeast lysate.

14. Add 5 mL of SD-URA medium into round-bottom test tubes for each positively identified transformant and add the corresponding patched yeast from the SD-URA/agar plate from step F1. Incubate at 30 °C overnight with vigorous shaking.

**H. Isolation of total yeast DNA and transformation into bacteria (~Day 10)**

This total yeast DNA isolation procedure (to isolate the circular HCoV-OC43-YCpBAC plasmids) is a modified version of the “DNA purification from yeast” protocol from the discontinued Gentra Puregene Yeast/Bact. kit. It involves a spheroplasting procedure similar to steps D5–D6, followed by the QIAGEN protocol from the cell lysis step to the RNase A treatment step. Once spheroplasts are made, lysis of the yeast should be easy, and alternative DNA isolation procedures should yield comparable results. Similar alcohol precipitation-based yeast DNA isolation kits are available from Thermo Scientific (Yeast DNA Extraction Kit, catalog number: 78870) and Zymo Research (Zymoprep Yeast Plasmid Miniprep I, catalog number: D2001). Since the goal of this procedure is to isolate intact, circular HCoV-OC43-YCpBACs, care must be taken to avoid steps that may shear or damage these large circular plasmids (excessive or harsh pipetting, vortexing, or column-based DNA isolation procedures).

1. The next morning, prepare a glycerol stock of each culture containing 750 μL of yeast culture and 250 μL of 50% glycerol. Store at -80 °C.

2. Pellet the remaining culture volume in the test tube at 3,000× *g* for 10 min at 4 °C and discard the supernatant.

3. Add 2 mL of 1 M sorbitol solution and resuspend the pellet with vortexing. Pellet the yeast cells at 3,000× *g* for 5 min at 4 °C and discard the supernatant.

4. Add 1.5 mL of SPE solution to the yeast cell pellet and resuspend with vortexing. Add 12 μL of zymolyase solution (10 mg/mL) and 3 μL of β-mercaptoethanol solution, mix well, and transfer to a 1.5 mL tube. Incubate at 37 °C for 30 min.

5. Pellet the spheroplasts at 700× *g* for 5 min and discard the supernatant.

6. Add 600 μL of cell lysis solution (QIAGEN) and mix by pipetting gently.

7. Add 200 μL of protein precipitation solution (QIAGEN) and mix by inversion.

8. Incubate the samples on ice for 5 min to enhance the precipitation of proteins.

9. Pellet the precipitated proteins, cell debris, and unlysed cells at 16,000× *g* for 3 min at 4 °C.

10. Add 600 μL of isopropanol to a new 1.5 mL tube and pour the supernatant from step H9 into the tube.

11. Mix by inversion 50 times.

**Critical:** It is important to thoroughly mix the cell lysate supernatant with the isopropanol to maximize the precipitation of the DNA from the solution.

12. Pellet the precipitated nucleic acids at 16,000× *g* for 5 min at 4 °C.

*Note: The precipitate (containing DNA, RNA, and YCpBAC plasmid DNA) may be visible as a small, white pellet against the side of the 1.5 mL tube.*

13. Discard the supernatant and wash the pellet with 500 μL of 70% ethanol with inversion.

14. Pellet the precipitated nucleic acids at 16,000× *g* for 5 min at 4 °C.

15. Discard the supernatant and aspirate any remaining volume with a P200 pipette.

**Critical:** Do not over-dry the nucleic acid pellet. This will make it difficult to resuspend.

16. Resuspend the nucleic acids with 100 μL of DNA hydration solution (QIAGEN). Flick to mix.

17. Add 1.5 μL of RNase A solution (QIAGEN) and mix by flicking. Incubate the solution at 37 °C for 15–60 min.

18. Optional: Incubate the solution at 65 °C for up to 1 h to fully dissolve the DNA.

19. Measure the concentration of the DNA using a spectrophotometer. Typical DNA yields are >400 ng/μL.

*Note: Blank the spectrophotometer against a solution of DNA hydration solution containing RNase A.*

**Pause point:** Yeast DNA samples can be used immediately or stored at -20 °C until needed.

20. Remove electrocompetent Stbl3 *E. coli* from -80 °C storage and thaw on ice (see General note 7). Chill the number of electroporation cuvettes required on ice.

21. In a 1.5 mL tube, combine 60 μL of electrocompetent Stbl3 *E. coli* with 10 μL of yeast DNA from step H17 and incubate on ice for at least 5 min.

*Note: Electroporating a large volume (10 μL) of high-concentration yeast DNA is needed to transfer the low concentration of circular YCpBAC plasmids into the bacteria. Volumes >10 μL may further reduce the electroporation efficiency and could result in arcing.*

22. Pipette the chilled DNA/bacteria solution into the chilled electroporation cuvette.

23. Electroporate the bacteria in a Gene Pulser II Electroporation System with Pulse Controller PLUS module using 0.2 cm gap width cuvettes and the following settings: 2.5 kV, 25 μF, 200 Ω.

24. Immediately following the electroporation, transfer the electroporated bacteria using a gel loading tip into a 1.5 mL tube containing 60 μL of LB medium.

25. Incubate the electroporated bacteria in LB at 37 °C for 1 h with shaking.

26. Add all the electroporated bacteria to an LB/agar plate containing 17.5 μg/mL chloramphenicol (see General note 8).

27. Incubate the plate at 37 °C for 1–2 days.

*Note: Expect ≤ 5 transformants per electroporation reaction (see General note 7).*

**I. Preparation of HCoV-OC43-YCpBAC plasmids for sequencing and virus rescue (~Day 12)**

We used QIAfilter Plasmid Midiprep kits (QIAGEN) for preparing sequencing/transfection-grade YCpBAC plasmid DNA. This kit can purify plasmids up to 50 kbp in a relatively short time with the QIAfilter step. Regardless of the manufacturer or kit selected to isolate YCpBACs from *E. coli*, ensure that the kit can isolate large plasmids (HCoV-OC43-YCpBACs are up to 42.3 kbp). Additionally, follow any modifications to the procedure that are indicated for low-copy plasmids. After the *E. coli* are lysed, perform only gentle mixing to avoid shearing the large YCpBAC plasmids.

1. Pick a colony from the LB/chloramphenicol plate, inoculate an LB/chloramphenicol starter culture (5 mL), and incubate at 37 °C for 6–8 h.

2. Add 200 μL of starter culture to a 100 mL LB/chloramphenicol culture and incubate at 37 °C overnight with shaking.

**Critical:** YCpBAC plasmids are low-copy, so it is important to use 100 mL cultures (or the volume recommended by the kit) to have sufficient material to obtain useful quantities of plasmid DNA.

3. The next day, prepare a glycerol stock of each culture containing 750 μL of bacterial culture and 250 μL of 50% glycerol. Store at -80 °C.

4. Follow the procedure outlined in the manufacturer’s instructions to complete the purification of the YCpBAC plasmids.

*Note: Some modifications to this protocol that may help increase plasmid yields include doubling the volumes of buffers P1, P2, and P3, increasing the elution/precipitation volumes, and warming buffer QF to 65 °C.*

5. Resuspend the plasmid DNA pellet after isopropanol precipitation and ethanol washing in 100 μL of Buffer EB.

**Critical:** Do not over-dry the plasmid DNA pellet. This will make it difficult to resuspend.

6. Measure the concentration of the plasmid DNA using a spectrophotometer blanked against buffer EB. Typical DNA concentrations are > 100 ng/μL.

**Critical:** It is strongly recommended to submit the plasmid DNA for whole plasmid sequencing (i.e., Plasmidsaurus) to ensure the sequence is free from unwanted mutations.

7. If planning to initiate the virus rescue the next day, incubate a 6-well plate with 2 mL/well of 5 μg/mL poly-L-lysine (diluted from 1 mg/mL in PBS) for 15 min to improve adherence of the 293T cells. Count the cultured 293T cells using a hemocytometer with trypan blue staining. Seed the 293T cells at a density of 2.5 × 10^6^ cells/mL into these coated wells. The desired confluency for transfection the following day should be 70%–80%.

**J. Transfection of plasmid DNAs to rescue recombinant HCoV-OC43 (~Day 13)**

As noted above, this reverse genetics system is designed for rescuing viruses from transfected cells by virtue of the mammalian transcriptional control elements built into the HCoV-OC43-YCpBACs (Figure 2). We have optimized the rescue to involve an initial transfection into the highly transfectable human embryonic kidney 293T (293T) cell line, followed by a co-culture with more permissive BHK-21 cells to facilitate virus production. We have not had success with direct transfection into baby hamster kidney 21 (BHK-21) cells; however, this may be possible with further optimization of the transfection efficiency. Additional mammalian cell lines may also be useful for the rescue of recombinant HCoV-OC43; however, transfectability and their ability to support viral replication would need to be determined. Co-transfecting a plasmid that encodes OC43-N with the HCoV-OC43-YCpBAC into the 293T cells will increase the efficiency of rescuing infectious viruses, as demonstrated for other coronavirus reverse genetics systems [4,39,40]. The following work should be performed in a biosafety cabinet using sterile technique. 293T cells were grown in DMEM supplemented with heat-inactivated 10% FBS, 100 U/mL penicillin, 100 μg/mL streptomycin, and 2 mM L-glutamine (Pen/Strep/Gln). BHK-21 cells were maintained in DMEM supplemented with 5% FBS and Pen/Strep/Gln. All cells were maintained at 37 °C in a 5% CO_2_ atmosphere.

1. The day prior to initiating the virus rescue, seed a 6-well plate of 293T cells onto poly-L-lysine-coated wells (see step I7).

2. Prepare the DNA solution for transfection containing 0.9–3.6 μg of HCoV-OC43-YCpBAC and 0.1–0.4 μg of OC43-N-encoding plasmid (pGBW-m4134906) diluted in Opti-MEM I reduced serum medium up to 100 μL of final volume (see General note 9). Also, prepare a negative control transfection with another plasmid (equivalent total DNA amount) to control for the impact of transfection on cell viability.

*Note: When monitoring for rescue of infectious virus, the cytopathic effect (CPE) may be an early indicator of the presence of infectious virus. However, with the long period in culture needed for rescue, plasmid DNA transfection alone may impact the health of the cells. Therefore, it is important to have a comparison (negative control transfection) to assess the health of the cells during the rescue, especially in the absence of a fluorescent reporter gene.*

3. Prepare the PEI solution containing 3 μL of 1 mg/mL PEI per μg of DNA diluted in Opti-MEM I reduced serum medium up to 100 μL of final volume.

4. Combine the DNA solution and PEI solution together and incubate at room temperature for 15–20 min to form the transfection complexes.

5. During the incubation in step J4, remove the growth medium from the 293T cells and replace with 1.3 mL/well of Opti-MEM I reduced serum medium.

6. After the incubation in step J4, gently add the transfection complexes dropwise into the medium covering the 293T cells.

7. Place the transfected cells in a 37 °C/5% CO_2_ incubator for 2–4 days.

**K. BHK-21 co-culture with transfected 293T cells to rescue recombinant HCoV-OC43 and virus passaging (~Day 15)**

1. Remove the medium from the transfected 293T cells and wash the cells gently with PBS. Trypsinize the transfected 293T cells from step J7 with 0.25 mL of trypsin/EDTA. Resuspend the cells in 0.75 mL of DMEM+2.5% FBS/Pen/Strep/Gln and seed into a 10 cm^2^ dish. Seed diluted BHK-21 cells (~1 × 10^6^ cells) in DMEM+2.5% FBS/Pen/Strep/Gln in the dish and make up the volume to 10 mL.

2. Leave the cells for 4–6 days at 37 °C/5% CO_2_ until CPE forms.

*Notes:*

*1. As the co-culture progresses, the 293T cells will round up and detach from the surface of the dish. The BHK-21 cells will remain mostly adherent even as CPE progresses.*

*2. Using a fluorescent reporter virus greatly assists in monitoring the progression of the virus rescue. When using a reporter virus, the 293T cells will become fluorescent first and will continue to produce large amounts of reporter protein even after they detach from the dish. As the infection proceeds in the BHK-21 cells, these cells will progressively accumulate more reporter protein and will emit brighter fluorescence. The expression of the reporter protein will precede the formation of CPE by 12–24 h.*

3. When CPE covers the monolayer, remove the medium from the cell monolayer and clear by centrifugation at 500× *g* for 5 min. This supernatant (passage 1) will be used to infect a 175 cm^2^ flask of BHK-21 cells to passage the virus.

**Pause point:** The virus-containing supernatant can be stored at -80 °C.

4. Infect a near confluent monolayer of BHK-21 cells in a 175 cm^2^ flask with 2.5 mL of passage 1 supernatant diluted with 7.5 mL of DMEM. Infect the cells at 37 °C for 1 h with rocking every 15 min.

*Note: If the titer is determined for the passage 1 supernatant, infect the BHK-21 monolayer with a multiplicity of infection of 0.1.*

5. Remove the inoculum and replace with 25 mL of DMEM+1% FBS/Pen/Strep/Gln.

6. Leave the cells for 2–3 days at 37 °C/5% CO_2_ until complete CPE.

7. When CPE covers the monolayer, remove the medium from the cell monolayer and clear by centrifugation at 500× *g* for 5 min. This supernatant (passage 2) will be used to infect a 175 cm^2^ flask of BHK-21 cells to passage the virus.

**Pause point:** The virus-containing supernatant can be stored at -80 °C.

8. Determine the titer of the recombinant HCoV-OC43 using a TCID50 assay.

9. Infect two near confluent monolayers of BHK-21 cells in 175 cm^2^ flasks at a multiplicity of infection of 0.1 with passage 2 supernatant diluted with DMEM (up to 10 mL of final volume per flask). Infect the cells at 37 °C for 1 h with rocking every 15 min.

10. Remove the inoculum and replace with 25 mL of DMEM+1% FBS/Pen/Strep/Gln.

11. Leave the cells for 2–3 days at 37 °C/5% CO_2_ until complete CPE.

12. When CPE covers the monolayer, remove the medium from the cell monolayers, pool together, and clear by centrifugation at 500× *g* for 5 min. This supernatant (passage 3) will be the working stock of the rescued virus. Store the virus in 0.5–1 mL aliquots at -80 °C.

## Validation of protocol

This protocol was used to successfully generate plasmids encoding recombinant wild-type and reporter HCoV-OC43 viruses. The DNA sequences of the four assembled plasmids are available on GenBank using the following accession numbers: CMVn-OC43-WT-Ribo-BGH-YCpBAC (PQ791637), CMVn-OC43-mClover-Ribo-BGH-YCpBAC (PQ791638), CMVn-OC43-mRuby-Ribo-BGH-YCpBAC (PQ791639), and CMVn-OC43-mCardinal-Ribo-BGH-YCpBAC (PQ791640). Infectious HCoV-OC43 viruses were rescued from these plasmids through transfection of HEK 293T cells followed by co-culture with BHK-21 cells. The rescued viruses were propagated in BHK-21 cells to generate viral stocks for subsequent experiments. Analogous to the general overview provided in the protocol above (Figure 1A), this protocol was used to insert either *mRuby3-H2B, mCardinal*, or *EBFP3* into our existing HCoV-OC43-YCpBAC plasmids [2]. The data below demonstrate the fitness of the recombinant, yeast-assembled (^YA^) OC43-mRuby^YA^ and OC43-mCardinal^YA^ viruses relative to HCoV-OC43 VR-1558 (ATCC; wild type) and the previously characterized OC43-mClover^YA^.

**Figure 6.**
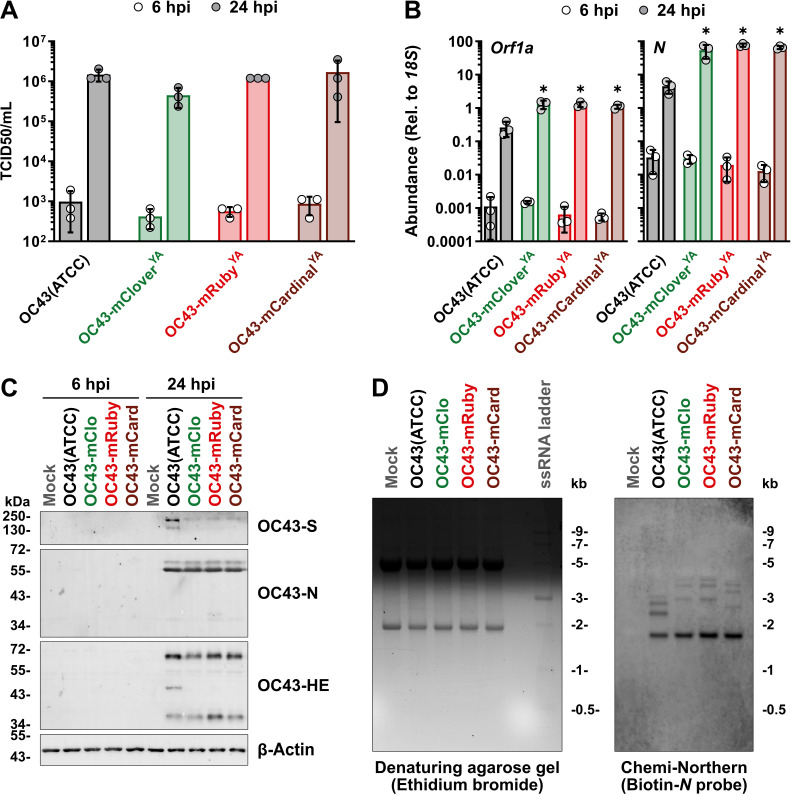
Yeast-assembled HCoV-OC43 reporter viruses replicate to comparable titers as wild-type HCoV-OC43 and produce an eighth sgRNA. (A) HEK 293A cells infected at an MOI of 0.1 for 1 h in serum-free medium with the indicated viruses, followed by a medium change to 2.5% DMEM+PSQ prior to collection of the supernatants at the indicated times. The virus-containing supernatants were titred on BHK-21 cells with five-fold serial dilutions using the TCID50 (Spearman-Kärber [41]) method. Data were plotted as the mean ± standard deviation from three biological replicates. No significant differences were obtained using two-way ANOVA (Tukey). (B) Total RNA from cells infected as in panel A was isolated using the RNeasy Plus Mini Kit (QIAGEN) and reverse transcribed using Maxima H Minus First Strand cDNA Synthesis Kit (Thermo Fisher) with random hexamers and oligo (dT)18 primers. qPCRs were performed using Luna Universal qPCR Master Mix (NEB) in 10 μL reactions with 200 nM primers [forward (Orf1a/N): 5′ CCGCTTCACTGATCTCTTG, reverse (Orf1a): 5′ ACCACTATGAAAAATCTACGCC, reverse (N): 5′ GAGGACGCTCTACTACTGG, forward (18S): 5′ TTCGAACGTCTGCCCTATCAA, reverse (18S): 5′ GATGTGGTAGCCGTTTCTCAGG] and 1:600 diluted cDNA. Analysis was performed using an efficiency-corrected 2−ΔΔCq method [42] with the level of expression of Orf1a (left panel) or N (right panel) relative to 18S plotted as the mean ± standard deviation from three biological replicates. Statistical significance was determined using two-way ANOVA (Tukey) and *P ≤ 0.0001. (C) Lysates from cells infected as in panel A were prepared with 2× Laemmli buffer containing dithiothreitol/bromophenol blue, and 5 μg per lane was separated on 10% acrylamide gels prior to transfer to polyvinylidene fluoride membranes using the Trans-Blot Turbo RTA Midi 0.2 μm PVDF Transfer Kit (Bio-Rad) and a Trans-Blot Turbo Transfer System (Bio-Rad). 4% bovine serum albumin in Tris-buffered saline containing 0.1% Tween-20 was used for membrane blocking and antibody incubations. The following antibodies were used: Mouse anti-OC43-N (1:2,500); rabbit anti-OC43-S (1:2,000); rabbit anti-OC43-HE (1:500); rabbit anti-β-Actin (1:1,000), goat anti-mouse IgG, Alexa Fluor 555 (1:10,000); goat anti-rabbit IgG, Alexa Fluor 647 (1:10,000); and goat anti-rabbit IgG, Alexa Fluor 488 (1:10,000). (D) Total RNA samples from cells infected as in panel A were isolated using the RNeasy Plus Mini Kit (QIAGEN) and separated on a 1% denaturing agarose gel containing ethidium bromide also loaded with a ssRNA ladder (NEB) (left panel). Following alkaline transfer and UV cross-linking of the RNA, the RNA blot was incubated with 25 nM biotinylated oligo probe (5′ Biotin-GCCTCATCGCTACTTGGGTCCCGATCGACAATGTCAGCCGGGGT) in hybridization solution overnight at 42 °C. Streptavidin-HRP (1:2,500) diluted in Odyssey Blocking Buffer (LI-COR) containing 1% sodium lauryl sulfate was incubated with the blot for 1 h at room temperature prior to detection using Clarity Max Western ECL Substrate (Bio-Rad) and a ChemiDoc MP Imaging System (Bio-Rad) (right panel). Abbreviations: Chemi, chemiluminescence; HE, hemagglutinin esterase; hpi, hours post-infection; HRP, horseradish peroxidase; kb, kilobases; kDa, kilodaltons; mCard, mCardinal; mClo, mClover; MOI, multiplicity of infection; N, nucleocapsid; Orf1a, open reading frame 1a; S, Spike; TCID50, 50% tissue culture infectious dose; YA, yeast-assembled.

To validate the functionality of the OC43-mRuby^YA^ and OC43-mCardinal^YA^ recombinant viruses, HEK 293A cells were infected with OC43(ATCC) or each of the three HCoV-OC43 reporter viruses (mClover^YA^, mRuby^YA^, or mCardinal^YA^) at a multiplicity of infection of 0.1 for 6 or 24 h. At the indicated times, infected cell supernatants were collected for assessing viral titers (using TCID50 assay; Figure 6A), infected cell lysates were prepared for RNA extractions and RT-qPCR performed on viral RNAs encoding *Orf1a* and *N* (Figure 6B), infected cell lysates were prepared for SDS-PAGE/western blotting to detect viral S, N, and HE proteins (Figure 6C), and Northern blotting was used to visualize the increase in sgRNA sizes produced by reporter viruses using RNA from infected cells (Figure 6D).

The amounts of infectious HCoV-OC43 reporter viruses released from infected cells were similar to the wild-type HCoV-OC43 [OC43(ATCC)] virus (Figure 6A). Consistent with our previous results with the OC43-mClover^YA^ virus [2], we observed that all three HCoV-OC43 reporter viruses produced larger quantities of viral genomic RNA (*Orf1a*) and sg-mRNA encoding *N* by 24 h post-infection when compared to OC43(ATCC) infection (Figure 6B). We previously speculated that these alterations in the accumulation of certain viral RNAs could be caused by altered transcriptional efficiency due to the insertion of reporter genes between the *M* and *N* loci [2]. When assessing viral protein production from these recombinant viruses, we could not detect viral proteins at 6 h post-infection, whereas abundant viral proteins were detected by 24 h post-infection (Figure 6C). Consistent with our previous findings [2], we observed that all viruses produced comparable levels of N and HE protein, but the reporter viruses produced less S protein (Figure 6C). These differences in S protein detection were also seen in our wild-type, yeast-assembled HCoV-OC43 (OC43^YA^) [2], indicating that the genotype of our strain may influence S protein production or its detection by western blot with the anti-OC43 S antibody. However, despite any differences in viral RNA or protein accumulation between the wild-type and reporter viruses, comparable levels of infectious virus were released from infected cells (Figure 6A). Owing to the 3′ co-terminal nature of coronavirus sgRNAs, sequence insertions will influence the length of any sgRNAs produced from TRSs upstream of the insertion. Using a chemiluminescence-based Northern blotting (Chemi-Northern) protocol, we observed the expected alteration in sg-mRNA migration due to our reporter gene insertions in OC43-mClover^YA^, OC43-mRuby^YA^, and OC43-mCardinal^YA^ (Figure 6D). The sensitivity of the chemiluminescent Northern blot using an anti-sense, biotinylated oligo probe (designed to nucleotides 469-512 of *OC43-N*) allowed for the detection of the four smallest sg-mRNAs produced by each virus (Figure 6D, right panel). The smallest sg-mRNA encoding *N* was the same size for all viruses (1.7 kb) (Figure 6D), as it is transcribed from a TRS downstream of the reporter gene insertions. The next three larger sg-mRNAs produced from TRSs upstream of the reporter gene insertions encode (in order of smallest to largest) *Rep, M*, and *E* (OC43-mClo, OC43-mRuby, OC43-mCard) and are visibly larger than the sg-mRNAs encoding *M, E*, and *NS5A* produced by OC43(ATCC) (Figure 6D). The sg-mRNAs produced by OC43-mClover^YA^ and OC43-mRuby^YA^ are similar in size and are shifted by 1,246 bases and 1,239 bases, respectively, compared to equivalent OC43(ATCC) sg-mRNAs. The sg-mRNAs produced by OC43-mCardinal^YA^ are only larger by 912 bases relative to comparable OC43(ATCC) sg-mRNAs due to the smaller reporter gene insertion in this virus (Figure 6D).

These data demonstrate the feasibility of using this TAR-based protocol for mutagenizing HCoV-OC43-YCpBACs and for using these plasmids to rescue infectious, recombinant HCoV-OC43 viruses.

This protocol (or parts of it) has been used and validated in the following research article(s):

• Duguay et al. [2]. A yeast-based reverse genetics system to generate HCoV-OC43 reporter viruses encoding an eighth subgenomic RNA. *Journal of Virology*.

## General notes and troubleshooting

**General notes**

1. Heat-inactivate restriction endonucleases if possible. Otherwise, clean-up or gel extraction is necessary to remove any endonucleases from the DNA solution prior to the TAR reaction. New England Biolabs DNA Cleanup/Gel Extraction kits are preferred for DNA purification steps as they isolate fragments up to 25 kb.

2. An alternative to using endonucleases to establish the site for mutagenesis is to perform PCR amplification to generate all the fragments needed for TAR. For practical reasons, restricting these amplicons to < 10 kb will make the PCRs easier to perform. Our preferred polymerase for long-range, high-fidelity PCRs is the KOD Xtreme Hot Start DNA Polymerase.

3. Determining these values allows one to prepare DNA solutions with appropriate molar ratios to favour the insertion of the HDR fragment(s) into the linearized YCpBAC. When using multiple fragments (3+) for assemblies, ensure that all non-YCpBAC fragments are present in equimolar ratios and are in excess of the YCpBAC fragment.

4. With yeast assembly reactions, the efficiency of assembly will decrease as the total number of fragments and/or the size (bp) of the fragments increase. Since the size of the HCoV-OC43 genome, the transcriptional control sequences, and the YCpBAC are fixed, trying to minimize the number of fragments in a TAR reaction is advised. If DNA amounts allow, performing increasing molar ratios of fragments to YCpBAC or different ng/pmol amounts of the YCpBAC may help in optimizing the assembly reactions.

5. Zymolyase activity can vary from lot to lot. It may be necessary to determine the appropriate volume of 10 mg/mL zymolyase solution needed for spheroplast preparation prior to yeast transformations or colony PCRs.

6. Salmon sperm DNA acts as a carrier and can improve spheroplast transformation efficiency [43]. Heat the salmon sperm DNA at 95 °C for 10 min before use and cool on ice.

7. We used electrocompetent Stbl3 *E. coli* for our work, which has mutations to reduce nonspecific recombination; however, other strains of *E. coli* used for large plasmid maintenance, such as DH10B (Thermo Fisher), 10-beta (NEB), or XL10 Gold (Agilent), are also suitable for propagating the YCpBAC plasmids. When transforming large plasmids, electrocompetent cells are recommended. At this stage, all *E. coli* transformants are typically correct; however, if a greater number of clones is desired, purchasing commercial electrocompetent *E. coli* could maximize the transformation efficiency.

8. Chloramphenicol is a translation inhibitor. While working concentrations are listed between 10 and 35 μg/mL, we prefer to use chloramphenicol at a final concentration of 17.5 μg/mL. This provides enough antibiotic to facilitate selection while decreasing the impact of translational inhibition on bacterial growth on plates or in liquid cultures.

9. For the transfection of 293T cells, it may be necessary to vary the amount of plasmid DNA used. We have successfully rescued infectious viruses using ratios of YCpBAC to OC43-N plasmid of 5:1, 6:1, and 9:1 with total DNA amounts of 1–4 μg.

**Troubleshooting**

Problem 1: Conversion to spheroplasts is not complete within 20–30 min.

Possible causes: Insufficient zymolyase solution added, lower-activity lot of zymolyase used, or β-mercaptoethanol has degraded.

Solutions: Incubate the reaction longer until the desired results are achieved. Consider adding 50%–100% more zymolyase solution on subsequent spheroplasting attempts. Prepare a fresh solution of β-mercaptoethanol from the 14.3 M stock solution.

Problem 2: Spheroplasts appear lysed in 1 M sorbitol solution.

Possible causes: Too much zymolyase used, the spheroplasting reaction was incubated too long, or shaking was too vigorous.

Solutions: Spheroplasts are fragile, and excessive incubation of yeast cells with zymolyase can lead to lysis of the spheroplasts. Reduce the amount of zymolyase or shorten the incubation time on subsequent attempts. Ensure the shaker is set to a low speed sufficient to gently mix the spheroplasting reaction.

Problem 3: High number of yeast background transformants in the “vector only” transformation.

Possible cause: Residual circular YCpBAC (undigested or PCR template) in the assembly reaction or recombination via non-homologous end joining.

Solution: For restriction-digested YCpBAC fragments, consider overnight digestion. For PCR-amplified YCpBACs, add *Dpn*I treatment following the PCR and cleanup using the Monarch Spin PCR & DNA Cleanup kit (NEB, T1130; for DNA up to 25 kbp). Adding less YCpBAC to the assembly reaction will also minimize background transformants.

Problem 4: Low or no bacterial transformants following yeast DNA electroporation.

Possible causes: (1) DNA concentration is too low, (2) DNA concentration is too high, (3) YCpBAC plasmids are damaged, (4) arcing during electroporation, (4) low transformation efficiency, or (5) wrong antibiotic used.

Solutions: (1) Perform additional electroporations using the same yeast DNA prep, (2) reduce the yeast DNA volume by half, (3) repeat the yeast DNA prep with care not to vortex or vigorously pipette the solutions after the zymolyase step, (4) ensure the *E. coli* used are highly competent or consider purchasing commercial electrocompetent *E. coli*, or (5) ensure chloramphenicol is used in the LB/agar plates (see General note 8, re: chloramphenicol). Simply repeating the yeast DNA electroporation into *E. coli* might yield colonies on subsequent attempts without alterations to the protocol.
